# Geodesign in the era of artificial intelligence

**DOI:** 10.1007/s44243-025-00054-5

**Published:** 2025-03-07

**Authors:** Xinyue Ye, Tianchen Huang, Yang Song, Xin Li, Galen Newman, Zhongjie Lin, Dayong Jason Wu

**Affiliations:** 1 Department of Landscape Architecture and Urban Planning & Center for Geospatial Sciences, Applications and Technology, Texas A&M University, College Station, TX 77840 USA; 2 Section of Visual Computing and Computational Media, School of Performance, Texas A&M University, Visualization, and Fine Arts, College Station, TX 77840 USA; 3 Weitzman School of Design, University of Pennsylvania, Philadelphia, PA 19104 USA; 4 Research & Implementation, Texas A&M Transportation Institute, Dallas, TX 75251 USA

**Keywords:** Geodesign, Artificial Intelligence, Ethics, Spatial planning

## Abstract

This paper explores the evolution of Geodesign in addressing spatial and environmental challenges from its early foundations to the recent integration of artificial intelligence (AI). AI enhances existing Geodesign methods by automating spatial data analysis, improving land use classification, refining heat island effect assessment, optimizing energy use, facilitating green infrastructure planning, and generating design scenarios. Despite the transformative potential of AI in Geodesign, challenges related to data quality, model interpretability, and ethical concerns such as privacy and bias persist. This paper highlights case studies that demonstrate the application of AI in Geodesign, offering insights into its role in understanding existing systems and designing future changes. The paper concludes by advocating for the responsible and transparent integration of AI to ensure equitable and effective Geodesign outcomes.

## Introduction

The use of innovative digital tools to analyze, design, and plan geographic space is often referred to as Geospatial Design (or Geodesign). Geodesign's early foundations were rooted in the 1960s development of Geographic Information Systems (GIS) (Tomlinson, 1969). Tomlinson’s work on the Canada Geographic Information System introduced a system for managing large, geographically referenced datasets (Barker, 2011). Harvard Laboratory for Computer Graphics and Spatial Analysis played a crucial role in the development of Geodesign by pioneering the use of computer technology in collaborative, data-driven design processes (Steinitz, 2016). Steinitz's "A Framework for Geodesign: Changing Geography by Design" (2012) presents a systematic approach to integrating geographic knowledge with design processes through six interconnected models (Representation, Process, Evaluation, Change, Impact, Decision) that emphasize collaboration, technology integration, iterative cycles, and ethical considerations to create sustainable and resilient environments. This framework underscores the iterative nature of design, where spatial data continually informs and refines design solutions (Steinitz, 2012).

McHarg’s Design with Nature (1969) transformed landscape architecture by promoting an ecological approach that integrates natural processes into design. His layered mapping of environmental factors like soil, vegetation, and hydrology laid the groundwork for modern GIS. Both McHarg and Steinitz applied Geodesign principles to urban and environmental challenges, building the Geodesign community through collaborative initiatives (McHarg, 1969; Steinitz, 2012). Geodesign combines geography with design methodologies, integrating systems thinking and digital technology for interdisciplinary planning (Goodchild, 2010; Steinitz, 2016). Its advancements in 3D modeling, AI, and urban analytics enhance data-driven decision-making and dynamic design processes (Campagna, 2016; Ervin, 2016; Wilson, 2015). This paper clarifies Geodesign's distinction from traditional spatial modeling and explores how AI enhances its effectiveness.

### Steinitz's framework of Geodesign

Steinitz's well-known work *"A Framework for Geodesign: Changing Geography by Design"* introduces six core questions that structure the Geodesign (Steinitz, 2012):

1. How should the study area be described?
  This question is about defining the characteristics of the area under consideration, covering physical, cultural, and environmental aspects. It aims to establish a clear understanding of the study area's current state.
2. How does the study area operate?
  This focuses on understanding the underlying systems, functions, and processes of the study area, such as ecological functions, social dynamics, or infrastructure performance.
3. Is the current study area working well?
  This question evaluates the current effectiveness of the study area in meeting its intended goals or sustaining its systems, identifying whether there are any existing issues or opportunities for improvement.
4. How might the study area be altered?
  The framework looks into possible changes or interventions that could be made, envisioning different scenarios or solutions for modifying the area.
5. What difference might the changes cause?
  This question assesses the potential outcomes and impacts of the proposed changes, predicting how the study area’s function, structure, or conditions might be affected.
6. How should the study area be changed?
  Finally, the framework calls for a decision on the best course of action, integrating all previous considerations to determine how to effectively implement the proposed changes.

The questions in Steinitz’s framework can be grouped into two categories: "Understanding the Existing System" and "Designing Future Changes." The first three questions focus on analyzing and evaluating the current conditions and performance of the study area, aiming to build a solid foundation of knowledge before any design work begins. The remaining three questions are forward-looking, concentrating on proposing, evaluating, and deciding on potential interventions to improve or modify the study area. Table 1 shows existing methods to deal with these issues in geodesign.

**Table 1:** Existing methods in Geodesign, and their strengths and limitations

Category	Method	Strengths	Limitations
Understanding the Existing System	Overlay Mapping	Visually represents complex environmental data, helps identify areas for development or conservation	Static nature, outdated/inaccurate data can lead to misguided decisions, cannot capture dynamic changes
	Site Analysis and Suitability Assessments	Provides a detailed understanding of the physical, biological, and cultural attributes of a site	Time-consuming, may not account for changing conditions or broader regional contexts
	Environmental Impact Assessment	Assesses environmental impacts and proposes mitigation measures, ensuring responsible development	Complex and resource-intensive, risk of bias, may underestimate cumulative impacts
Designing Future Changes	Physical Models and Hand-drawn Sketches	Facilitates communication and collaboration, helps visualize 3D aspects of designs	Labor-intensive, difficult to modify, lacks precision for large-scale or data-driven designs
	Evidence-based Design and Planning	Informs design through empirical, data-driven insights, aligns with Geodesign's scientific rationale	Relies on availability and quality of data, interpretation can be subjective, data may be outdated or scarce
	Participatory Design and Planning	Involves stakeholders in the design process, leverages local knowledge and perspectives	Time-consuming, difficulty reconciling diverse perspectives, risk of vocal groups dominating outcomes

For the first category of questions in “understanding the existing system”, existing methodologies such as overlay mapping, site analysis and suitability assessments, and environmental impact assessments are commonly used (Table 1). These approaches help to describe, analyze, and evaluate the current conditions of the study area in detail. Overlay mapping is a key element of Geodesign methods (McHarg, 1969), involving the layering of thematic maps like topography, vegetation, hydrology, and land use to provide a comprehensive landscape understanding (Goodchild, 2010; McHarg, 1969). This visual, systematic approach evaluates land suitability based on ecological principles, enabling planners to assess cumulative environmental impacts and make environmentally sensitive decisions (McHarg, 1969). Geodesign integrates proposals with impact simulations, using geographic contexts and systems thinking to foster collaboration between communities, designers, and scientists. (Steinitz, 2016). The strength of overlay mapping lies in its ability to visually represent complex environmental data, making it easier to identify areas suitable for development, conservation, or other land uses (Steiner, 2013). This method has been instrumental in promoting an ecological approach to planning, emphasizing the importance of respecting natural processes and patterns in design decisions.

Site analysis and suitability assessments are critical components of Geodesign (Reynolds, 2014). This process involves a detailed examination of a site's physical, biological, and cultural attributes to determine its suitability for various types of development or conservation (Ernst et al., 2019). This method requires a comprehensive understanding of the site's conditions such as its topography, soil, vegetation, climate, hydrology, as well as its socio-economic and cultural context. The goal of site analysis and suitability assessment is to identify the most appropriate, sustainable, and efficient use of land (Davis et al., 2021). This involves balancing the natural characteristics of the site with the needs and aspirations of the community. It is a process that requires careful consideration of various factors, including environmental constraints, potential impacts on biodiversity, and the capacity of the land to support different types of uses.

As noted, environmental impact assessments are also a critical process in Geodesign. These assessments involve evaluating the potential environmental impacts of proposed developments and identifying measures to mitigate negative effects (Campagna et al., 2019). This process is essential for ensuring that development projects are environmentally responsible and sustainable. It includes assessing the impact on ecosystems, biodiversity, water and air quality, and other environmental factors. It involves predicting and evaluating the likely environmental impacts, both positive and negative, and proposing mitigation measures to reduce adverse effects. The process typically includes several stages, such as screening, scoping, impact analysis, mitigation, reporting, and monitoring (Morgan, 2012). It requires a thorough understanding of the environmental baseline conditions and the potential changes that a proposed development might bring.

For the second category of questions in “designing future changes,” methods like physical models and hand-drawn sketches, evidence-based design and planning and participatory design and planning are more suitable (Table 1). These focus on developing, testing, and refining potential changes while involving stakeholders and leveraging empirical evidence to ensure that interventions are both effective and appropriate.

Physical models and hand-drawn sketches were essential in early Geodesign projects and processes (Moura, 2015). These methods provided tangible representations of spatial data and design proposals, facilitating communication and collaboration among designers, planners, and stakeholders. Physical models were particularly valuable for understanding the three-dimensional aspects of design proposals, allowing for a more intuitive grasp of scale, form, and spatial relationships (D. J. Lee et al., 2014; Steiner, 2013). Physical models and sketches played a crucial role in the design process, serving as tools for exploration, communication, and collaboration. They allowed designers and planners to test ideas, visualize concepts, and engage stakeholders in a more interactive and tangible way (Risinger, 2012).

Evidence-based design and planning places significant importance on using empirical data to facilitate design decisions. Evidence-based design is a fundamental principle in the field of Geodesign, which emphasizes the use of data-driven insights to inform and shape design decisions (Hamilton, 2003). This phenomenon may be seen via empirical research that examines the therapeutic benefits of green spaces on mental well-being, with the aim of providing insights for the design of urban parks (Lee & Maheswaran, 2011). Additionally, studies also explore the complex interplay between urban morphologies and community mobility (Lee & Maheswaran, 2011; Wang et al., 2020). Evidence based design and planning are highly aligned with Geodesign in that both approaches integrate a scientific rationale for decision making as well as assess intervention strategies through impact and performance modeling. Evidence-based designs and plans are inherently grounded in quantitative performance measures. Unfortunately, many of these performance measures, while increasing, have not yet been fully incorporated into Geodesign projects. Many measures have recently been developed through performance modeling which seek to more scientifically evaluate impacts and more accurately measure the effectiveness with which developed solutions fulfil their intended purpose. These measures and impact models are highly supported through the analytics and visualization capabilities made possible by AI.

Participatory design and planning, also known as co-design/planning, is based on the active involvement of stakeholders throughout the design process (Ernst et al., 2019; Sanders & Stappers, 2008). Steinitz (2012) highlighted the importance of Geodesign as a multidisciplinary approach to landscape and urban planning, emphasizing the need for collaborative, flexible, and adaptive methods to address complex environmental and urban challenges. Within the domain of Geodesign, it is essential to place significant attention on engaging stakeholders because of the broad and diverse nature of Geodesign projects. Stakeholders play a crucial role in the identification of possible difficulties and opportunities, as well as in the iterative assessment of design scenarios (Flaxman, 2010). Their richness of localized information and views makes them vital in this process. This paradigm aligns well with the fundamental principles of Geodesign, which promote an iterative and systems-oriented design philosophy (Steinitz, 2012). In addition, human-centered approach is characterized by its sympathetic approach, which places a high priority on addressing the needs, desires, and experiences of the targeted end-users (Norman & Stappers, 2015; Wu et al., 2023).

Geodesign, as a design philosophy, demands a thorough evaluation of every stakeholder involved, so guaranteeing that the results are not only practical but also culturally meaningful. The inclusion of participatory design techniques is often required, including the active engagement of stakeholders and the incorporation of their comments to enhance the refinement of design scenarios (Flaxman, 2010). This allows Geodesign solutions to undergo comprehensive assessments that go beyond considering just physical or environmental characteristics. This is especially beneficial when integrating datasets involving citizen science, the practice of collecting and analyzing data related to the natural world by members of the general public, into the Geodesign process.

## Limitations of existing methods in Geodesign

Existing Geodesign methods, while valuable in planning and design, have limitations (Flaxman, 2010; McHarg, 1969; Steinitz, 2012). Understanding these limitations is crucial for advancing the practice of Geodesign and addressing contemporary challenges. First, overlay mapping, though visually useful, is static and struggles to reflect dynamic ecological and social systems, especially temporal heterogeneities (Tulloch, 2017). Additionally, outcomes depend on accurate data, and outdated or incorrect data can lead to misguided decisions (Fusco et al., 2017). Traditional site analysis is often time-consuming and may not account for rapidly changing environments (Campagna & Di Cesare, 2016). Further, methods may focus too narrowly on local contexts, neglecting broader regional implications (Steinitz, 2012). In addition, environmental impact assessments are complex, requiring multidisciplinary input, and often overlook cumulative impacts, risking underestimation of true environmental effects (Therivel, 2013).

Physical models and hand-drawn sketches, while useful for visualizing spatial relationships and engaging stakeholders, are time-consuming and labor-intensive, making frequent modifications difficult. They also lack the detail and precision needed for large-scale or data-driven designs, limiting their support for quantitative analysis and informed decision-making compared to digital tools.

Relatedly, while participatory design seeks to engage the community actively, it encounters difficulties in accommodating the diverse viewpoints and interests present. The process tends to be lengthy and is frequently subject to disputes or delays in decision-making due to the challenge of reconciling various stakeholder perspectives. Moreover, there exists a risk that vocal groups may overshadow the contributions of less vocal participants, potentially skewing outcomes. This issue largely stems from disparities in resources and organizational capabilities among stakeholders, complicating the achievement of equitable and inclusive design solutions (Ortega Sánchez de Lerín, 2019; Sanoff, 1999). Similarly, evidence-based design and planning relies on the availability and quality of research and data. In many cases, relevant data may be scarce, outdated, or not specific enough for particular design contexts. Moreover, the interpretation of data and research findings can be subjective, leading to different conclusions and design outcomes (Albert et al., 2021).

### Incorporation of Artificial Intelligence (AI) into Geodesign

AI integration into Geodesign enhances data analysis, predictive modeling, decision-making, and participatory processes, surpassing traditional methods (Du et al., 2023a, 2023b; Ervin, 2016; Ye et al., 2021). It revolutionizes spatial data interpretation, efficiently handling large datasets and identifying complex spatiotemporal patterns (Liu et al., 2023; Mortaheb & Jankowski, 2023; Tang et al., 2020; Wang et al., 2016; Wu et al., 2022). Deep learning is a subset of AI that involves training neural networks with many layers to automatically learn patterns and representations from large amounts of data (LeCun et al., 2015). Convolutional Neural Networks (CNNs), a popular deep learning model, are used for processing grid-like data, especially images, by identifying spatial features (Yamashita et al., 2018; Ye et al., 2022), and in Geodesign, they are applied to land cover classification and satellite image analysis for spatial planning (Fan et al., 2023; Zhang et al., 2019). Long Short-Term Memory (LSTM) networks, another deep learning method, are designed for sequential data and can capture long-term dependencies (Graves, 2012), making them ideal for predicting time series data like urban growth or environmental changes in Geodesign (Wurm et al., 2021). Recurrent Neural Networks (RNNs), while similar to LSTMs, retain short-term memory of previous inputs (Caterini & Chang, 2018), and are useful for tasks such as analyzing traffic patterns and environmental changes over time, though they are limited in capturing long sequences (Lukic Vujadinovic et al., 2024). Transformers, an advanced deep learning model, use attention mechanisms to handle sequential data without relying on recurrence (Lin et al., 2022), making them powerful for integrating large-scale, complex geospatial datasets or automating design tasks in Geodesign, such as interpreting geographic features from text descriptions (Deng et al., 2024).

Generative AI refers to a subset of deep learning that focuses on generating new content, such as images, text, or other data, by learning patterns from existing datasets (Deng et al., 2024). It has gained significant attention and rapid development in recent years. some of the more popular generative AI models include Generative Adversarial Networks (GANs), consisting of a generator and discriminator that compete to create highly realistic outputs (Goodfellow et al., 2014), are applied in Geodesign to generate urban layouts or simulate environmental impacts (Huang et al., 2022; Ye et al., 2022). Variational Autoencoders (VAEs), which encode and sample from a latent space to generate diverse design options (Doersch, 2021), making them useful in Geodesign for exploring alternative landscape or urban scenarios (Xu et al., 2021). Additionally, diffusion models iteratively refine noise into structured images (Croitoru et al., 2023), making them effective for visualizing complex environmental phenomena like flood patterns (Shao et al., 2024). These generative AI models are transforming Geodesign by automating the generation of realistic design scenarios, enhancing creative exploration, and supporting data-driven decision-making in urban and environmental planning.

Multi-objective optimization is the process of optimizing two or more conflicting objectives simultaneously, often resulting in a set of solutions, where improving one objective means compromising another (Deb et al., 2016). Unlike deep learning, which focuses on learning patterns from large datasets for predictions or content generation, multi-objective optimization is about finding balanced solutions for competing goals. Methods such as Ridge Regression, which regularizes coefficients to handle multicollinearity (McDonald, 2009), are used in Geodesign to model spatial data, such as predicting environmental changes based on multiple correlated factors (Carneiro et al., 2022). Multiple Linear Regression models the relationship between a dependent variable and multiple predictors (Eberly, 2007), helping Geodesigners quantify how factors like land use and population affect urban growth (Triantakonstantis & Mountrakis, 2012). Logistic Regression predicts binary outcomes (LaValley, 2008), useful in Geodesign for categorizing areas as suitable or unsuitable for development (Siddiqui et al., 2018). Lastly, the Non-dominated Sorting Genetic Algorithm (NSGA-II) is an evolutionary algorithm that identifies optimal trade-offs in multi-objective problems (Yusoff et al., 2011), allowing Geodesigners to balance competing objectives like cost-efficiency and environmental sustainability in urban planning (Quan, 2019; Zhu et al., 2023). These methods help analyze and optimize geospatial data for more informed design decisions.

### Application of AI in Geodesign

As the two categories of six questions in Steinitz's framework of Geodesign, We can also classify the application AI in Geodesign into two categories: Understanding the existing system and designing future changes (Table 2). For the first category, AI has been widely applied in land se and land cover classification, urban heat island effect (UHI), energy consumption and resource use, and hazard risk and resilience assessment.

**Table 2:** AI applications in Geodesign and related cases

Category	Application Area	Case	Reference
Understanding the Existing System	Land Use and Land Cover Classification	FACNN applied to remote sensing images in Wuhan to classify and detect urban land cover changes, improving classification accuracy	(Zhang et al., 2019)
		HCRNN applied to multispectral Sentinel-2 data to classify land cover in Guangxi, achieving 97.62% accuracy	(Fan et al., 2023)
		Two-level machine learning model classifies urban forms in Taipei using multi-class classification and clustering models	(Chen et al., 2023)
	Urban Heat Island (UHI) Effect	CNN-based model used for building stock and heat demand analysis in urban areas, reducing heat demand by 47%	(Wurm et al., 2021)
		CNN downscaling of land surface temperature (LST) in Paris improves the representation of UHI and temperature extremes	(Johannsen et al., 2024)
		DNN models for UHI pattern prediction in Seoul, introducing UHI-hours metric to assess the long-term impact of UHI	(Oh et al., 2020)
	Energy Consumption and Resource Use	Data mining (clustering, PCA, RF) used to identify key features in building energy consumption, offering insights for energy-efficient design	(Shan et al., 2022)
		NSGA-II combined with parametric simulation to optimize energy use and comfort in old communities, significantly reducing emissions	(Z. Li et al., 2023)
		Energy estimation techniques reviewed to raise awareness in ML, encouraging energy-efficient practices in algorithm development	(García-Martín et al., 2019)
	Hazard Risk and Resilience Assessment	Hybrid CNN + SVM models applied for landslide susceptibility in Pakistan, improving prediction accuracy	(Aslam et al., 2021)
		DBN + PSO used for flood hazard mapping in Western Iran, achieving the highest predictive performance	(Satarzadeh et al., 2022)
		DNN models for predicting earthquake-triggered disaster chains in Sichuan, demonstrating high accuracy in cascading disaster risks	(Su et al., 2022)
Designing Future Changes	Scenario-Based Urban Design Generation	CAIN-GAN applied to generate context-sensitive urban designs in NYC, enhancing automated site planning with sustainability evaluation	(Jiang et al., 2024)
		Urban-GAN enables participatory urban design using GAN and case-based reasoning, generating designs for cities like Manhattan and Portland	(Quan, 2022)
		DNN models combined with urban planning knowledge to generate realistic, context-aware street networks in urban areas	(Fang et al., 2022)
	Optimization of Green Infrastructure	CA-Markov + MSPA applied to optimize UGI in Beijing, enhancing connectivity and sustainable urban development by 2030	(Ma et al., 2022)
		SVM + SHAP + NSGA-II used to plan GI for flood-prone areas in Beijing, optimizing flood prevention and investment efficiency	(Chen et al., 2024)
		MLP + SWMM meta-model used for urban stormwater management in Tehran, reducing runoff volume and pollution levels	(Raei et al., 2019)
	Sustainable Urban Planning	MDP + AI applied to optimize urban water resource management, improving water distribution and economic efficiency	(Xiang et al., 2021)
		Genetic algorithms combined with AIAD to co-evolve urban planning solutions in high-density areas like Gangnam, Seoul	(Quan et al., 2019)
		Deep learning applied to optimize public transport headways in Belgrade, improving regularity and passenger comfort	(Lukic Vujadinovic et al., 2024)
	Renewable Energy Infrastructure Design	AI and multi-criteria analysis applied to optimize energy use and solar radiation management in Georgia Tech campus	(Chang et al., 2019)
		ResNet + engineering simulations used to predict energy consumption in urban buildings in California, improving multi-scale energy predictions	(Nutkiewicz et al., 2018)
		MUZO optimization applied to high-rise buildings for energy efficiency in dense urban areas, meeting LEED standards	(Ekici et al., 2021)

AI has revolutionized land use and land cover classification, improving accuracy in detecting changes in urban and natural landscapes. For instance, Zhang et al. (2019) used a fully Atrous convolutional neural network (FACNN) to classify land cover in Wuhan, China, outperforming other models. Similarly, Chen et al. (2023) introduced a hierarchical convolutional recurrent neural network (HCRNN) for multispectral remote sensing, achieving high classification accuracy in forest cover monitoring. AI is also pivotal in assessing the urban heat island (UHI) effect, with deep learning models improving predictions of UHI patterns (Johannsen et al., 2024; Oh et al., 2020). In energy optimization, AI-driven methods like those by Shan et al. (2022) and Li et al. (2023) have enhanced energy efficiency and comfort in buildings. These tools leverage data mining and optimization algorithms to identify key features influencing energy use. Additionally, AI's role in hazard risk assessment has improved disaster preparedness, with hybrid models combining CNN and traditional methods to predict landslides (Aslam et al., 2021) and floods (Satarzadeh et al., 2022). AI also supports sustainable urban planning and design, generating context-aware designs through frameworks like CAIN-GAN (Jiang et al., 2024) and Urban-GAN (Quan, 2022). In renewable energy infrastructure, AI techniques optimize performance, such as Chang et al. (2019) using reinforcement learning for sustainable campus design, while Nutkiewicz et al. (2018) employed machine learning to model energy consumption. Lastly, AI aids in optimizing high-rise building designs, meeting energy efficiency standards like LEED (Ekici et al., 2021).

## Challenges in Integrating AI in Geodesign

The integration of AI into Geodesign heralds a new era of spatial planning and design, offering transformative potential in how we approach urban and environmental challenges. However, this integration is not without complexities and limitations. Understanding these challenges is crucial for leveraging AI effectively in Geodesign while mitigating potential drawbacks.

One of the primary challenges in incorporating AI into Geodesign is the heavy reliance on data. AI algorithms, particularly ML models, require large volumes of high-quality, accurate data to function effectively. However, acquiring such comprehensive data can be challenging, especially in less developed regions where data may be scarce or non-existent (Dehghan Hosseinabadi, 2018). Further, data quality issues such as inaccuracies, inconsistencies, and biases can lead to flawed AI analyses and predictions, potentially resulting in suboptimal design decisions (Du et al., 2023a, 2023b). This reliance on data is somewhat of a double-edged sword; while it enables AI to process complex information, it also makes AI as good as the data it is fed.

Another significant challenge is the complexity and interpretability of AI models. Many AI algorithms, especially those based on DL, are complex and difficult in understanding how these algorithms arrive at specific conclusions or predictions. This lack of transparency can be problematic in Geodesign, where stakeholders often require clear explanations for design decisions (Bishop, 2013; Ervin, 2016). This opacity complicates determining responsibility for outcomes, especially if negative impacts arise. Additionally, transparency and interpretability is essential for the iterative improvement of AI models. Understanding how decisions are made allows developers and users to identify shortcomings, errors, or areas for enhancement in AI systems. Without this insight, improving the accuracy, efficiency, and relevance of AI applications in geodesign becomes a much more difficult task. (J. Du et al., 2023a, 2023b).

The risk of over-reliance on AI in Geodesign is also a concern that cannot be overlooked as an excessive dependence on AI might diminish the role of human expertise and judgment in Geodesign and stifle human creativity and innovation in the design practices. AI should augment, not replace, the unique insights and experience of professionals. There have also been cases in which AI algorithms inadvertently perpetuated biases and led to unfair outcomes. Such a risk raises ethical concerns in their application into Geodesign processes (Safdar et al., 2020).

There are growing concerns over ethical use of data and privacy issues in the use of AI in Geodesign (Safdar et al., 2020). Firstly, the extensive data needed for AI training, especially for generative AI large models, poses a heightened risk to data security, as it encompasses a wider range of personal information. Secondly, AI's output, derived from complex processing, subtly alters input data, making privacy breaches less detectable and more covert compared to previous more straightforward digital tool operations (Peltz & Street, 2020). Geodesign often involves sensitive data, including information about individuals and communities, especially when integrating citizen science. Thus, ensuring the privacy and security of data in AI applications is paramount (Kamila & Jasrotia, 2023; Peltz & Street, 2020).

Finally, we are also facing technological and resource constraints. The cost of developing, implementing, and maintaining AI systems can be prohibitive, especially for smaller organizations or projects. Managing AI systems in Geodesign projects requires specialized skills, which may not be readily available in many organizations. Hence, the use of AI in Geodesign emphasizes the need for substantial technological infrastructure and expertise for effective AI integration. In general, AI algorithms perform better in specific tasks and fall short in adaptability and flexibility often required by some Geodesign projects. This means human intuition and experience must step in to solve some ambiguous or complex problems in reality.

### Future prospects and ethical considerations

The integration of AI into Geodesign represents a new phase of this evolving practice. In the face of significant technological advancements, adopting a mindset that combines enthusiasm for innovation with a critical understanding of its implications is imperative.

First, the potential of AI in transforming Geodesign is immense. AI’s ability to process and analyze large datasets can lead to better informed decision-making in urban planning and environmental management. AI can enhance the efficiency and accuracy of spatial planning, offering innovative solutions to complex problems (Batty, 2018; Yigitcanlar et al., 2020). However, this advantage comes with some tradeoffs. AI systems, particularly those based on DL, can be complex and often operate as “black boxes.” This lack of transparency can be problematic in Geodesign, where understanding the rationale behind design decisions is crucial (Kitchin, 2016). The integration of AI should not be seen as a replacement for human expertise but as a tool to amplify it. While AI can automate and optimize various aspects of Geodesign, it cannot replace the nuanced understanding and creativity of human professionals. This balance is crucial for ensuring that Geodesign remains a human-centered discipline, grounded on ethical and sustainable practices (Ye et al., 2023a). These ethical considerations are paramount when using AI in Geodesign. Concerns regarding data privacy, algorithmic transparency, and fair outcomes should take precedence in discussions surrounding AI integration (Sanchez et al., 2024). Ensuring that AI is used responsibly and ethically is essential for maintaining public trust and achieving equitable and sustainable results (Ye et al., 2023b). Finally, the field of Geodesign will continue to evolve, thanks to the advancement of AI and other digital technologies (Ye et al., 2023a). This means professionals in this field should commit to learning and adaptation, staying abreast of these new tools and techniques in order to produce best practices. This commitment is essential for harnessing the full potential of AI in Geodesign.

## Conclusion

The integration of AI into Geodesign marks a transformative step in how we approach urban planning, environmental management, and spatial design. This paper has explored how AI enhances traditional Geodesign processes by offering advanced tools for data analysis, predictive modeling, and design generation. Applications of AI, such as land use classification, energy consumption optimization, and green infrastructure planning and scenario-based urban design generation have demonstrated the potential of AI to improve the accuracy and efficiency of planning processes. However, the challenges associated with AI in Geodesign—such as data quality, model transparency, and ethical considerations around privacy and bias—require ongoing attention.

AI's role in Geodesign is not to replace human expertise but to augment it, providing a more nuanced and data-driven approach to tackling complex urban and environmental challenges. As AI technologies continue to evolve, they will play an increasingly important role in shaping sustainable and resilient cities. Future research should focus on addressing the limitations of AI models, particularly in terms of transparency, ethics, and inclusivity, ensuring that the benefits of AI-driven Geodesign are distributed equitably. In conclusion, while AI has opened new possibilities for Geodesign, a balanced approach that integrates technological innovation with human oversight and ethical practices is essential for creating sustainable, resilient, and inclusive urban environments.

## Data Availability

Data from this project is available upon request.
